# Vaccine-hesitant people misperceive the social norm of vaccination

**DOI:** 10.1093/pnasnexus/pgad132

**Published:** 2023-04-15

**Authors:** Eva Vriens, Luca Tummolini, Giulia Andrighetto

**Affiliations:** Institute of Cognitive Sciences and Technologies, Italian National Research Council, Via S. Martino della Battaglia 44, 00185 Rome, Italy; Institute for Futures Studies, Holländargatan 13, 11136 Stockholm, Sweden; Institute of Cognitive Sciences and Technologies, Italian National Research Council, Via S. Martino della Battaglia 44, 00185 Rome, Italy; Institute for Futures Studies, Holländargatan 13, 11136 Stockholm, Sweden; Institute of Cognitive Sciences and Technologies, Italian National Research Council, Via S. Martino della Battaglia 44, 00185 Rome, Italy; Institute for Futures Studies, Holländargatan 13, 11136 Stockholm, Sweden

**Keywords:** social norms, false consensus, norm diagnosis, norm interventions, vaccine hesitancy

## Abstract

Vaccine hesitancy is one of the main threats to global health, as became clear once more during the COVID-19 pandemic. Vaccination campaigns could benefit from appeals to social norms to promote vaccination, but without awareness of the social norm in place any intervention relying on social norms may backfire. We present a two-step approach of social norm diagnosis and intervention that identifies both whether a vaccination norm exists or develops over time and corrects misperceptions. In two studies (N=887 and N=412) conducted in Rome, Italy from June to August 2021 (during the first COVID-19 vaccination campaign), we show that vaccine-hesitant people strongly underestimated vaccine acceptance rates for COVID-19 despite increases in region-wide vaccination rates. This suggests a false consensus bias on the social norm of vaccination. We presented a subgroup of vaccine-hesitant people with the accurate vaccine acceptance rates (both planned uptake and vaccine approval) and tested if this social information would lower their vaccine hesitancy. We do not find clear effects, most likely because of the introduction of the COVID-19 health certificate (the “green pass”) that was implemented during our data collection. The green pass reduced both misperceptions in the social norm and vaccine hesitancy, thus undermining our treatment effect. We conclude that to alleviate misperceptions on the social norm of vaccination in early stages of the vaccination campaign governments and media should report not just the current vaccination rate, but also about vaccination intentions and approval.

**Significance Statement**

We explored whether a social norm of vaccination emerged during the COVID-19 vaccination campaign and found that this norm was slow to develop. Vaccine-hesitant people strongly underestimated the rate of vaccine acceptance and the appropriateness of vaccinating, suggesting that norms were likely of little help to increase vaccination rates. We illustrate how information about social norms can be used as part of public vaccination campaigns, but do not find clear support for our hypothesis that such messages would reduce vaccine hesitancy. The COVID-19 health certificate that was introduced during our data collection decreased both the misperception of the descriptive social norm and the vaccine hesitancy and as such likely replaced the effect of our norm-based interventions.

## Introduction

In 2019, the World Health Organization identified vaccine hesitancy—vaccine refusal or delayed vaccine acceptance (1)—as one of the top 10 threats to global health (2). This has only been exacerbated by the COVID-19 pandemic, which illustrated once more how important vaccines are to contain the spread of contagious diseases and prevent excess mortality rates. Like the measles virus and the human papillomavirus, the coronavirus responsible for COVID-19 threatens public health worldwide as long as high vaccination rates are not reached (3). Two major challenges were thus to distribute vaccines equally worldwide (4) and to decrease vaccine hesitancy (5, 6), for even in countries where COVID-19 vaccines were widely available, vaccine acceptance rates remained far below the estimates needed to effectively control the pandemic (7, 8).

Vaccine hesitancy is often ascribed to individual beliefs and preferences, such as trust (in the vaccine or provider) and complacency (perceived need for a vaccine) (9). Hence, with the start of COVID-19 vaccination programs, campaigns in most countries informed about safety and effectiveness or the possibility to return to normal life (10, 11), provided nudges and reminders to help people follow through on making their appointments (12, 13), used monetary incentives to get vaccinated (14) and implemented COVID-19 health certificates that restricted access to public spaces (15, 8).

Vaccine hesitancy is, however, also influenced by social factors, and many experts have advised to leverage the power of social norms to increase vaccination rates (16–19). Indeed, social norms play an important role in promoting public health (20, 21). With respect to vaccination in particular, earlier studies found social norms promoting vaccination to be positively related to vaccination intentions and uptake for influenza (22–25), human papillomavirus (HPV) (26–28), and COVID-19 (29–32).

Social norms are informal and shared behavioral rules that prescribe what people should or should not do (33–35). According to an influential account (33), people perceive social norms through expectations about the behavior of others as well as their estimated normative attitudes (empirical and normative expectations) and choose to act on these norms if both expectations are sufficiently high. Prominent approaches in psychology and economics often make a distinction between these two aspects in the form of descriptive norms (what others do) and injunctive norms (how most people think one ought to act) (34), but social norms are most effective when both descriptive and injunctive components are present at the same time (33).

This combined effect is particularly important in the case of vaccination. Even if vaccination is typically construed as an individual decision-making problem in which the risks from disease infection are weighted against the possible harms from the vaccine side effects, it is important to realize that vaccination reduces the transmission rate of the disease (36). One person’s decision to vaccinate thus bears a fundamental positive externality on others. Achieving high vaccination rates therefore constitutes a social dilemma in which individual and collective interests may conflict (37, 38). As for other social dilemmas (39), social norms (containing a descriptive and an injunctive component) may provide an important mechanism to foster cooperation.

We define empirical expectations about the vaccination norm as people’s perception of how many others (want to) take the vaccine (the descriptive component). Normative expectations concern people’s beliefs about how appropriate vaccinating is in the eyes of the others (the injunctive component). In the case of vaccination, relying on empirical expectations alone may not be sufficient. Since higher vaccination rates in the population (or widespread natural immunization) lower the risk of infection at the individual level, relying on high empirical expectations about vaccination rates can in fact be self-defeating and might lead instead to an (unintended) increase in vaccine hesitancy (40). If people believe that most others are already vaccinated or plan to do so, they may free-ride on those decisions and opt out of vaccinating themselves. Therefore, it may be hypothesized that social norms will be able to effectively promote vaccination when high empirical expectations about vaccination rate are coupled with the formation of appropriate normative expectations about this decision, which include expectations of social sanctions.

At the same time, an injunctive norm about the appropriateness of vaccinating that is not coupled with the belief that most others (plan to) get vaccinated is likewise ineffective (41), since in these situations people may realize that norms can easily be violated without sanctions (42). Instead, when both descriptive and injunctive aspects work in the same direction their combined effect can be sufficiently motivating (43).

While it is reasonable to expect that also in the case of COVID-19 social norms may have been important drivers for vaccination decisions, it should be noted that social norms about COVID-19 vaccination developed in a period characterized by uncertainty. Knowledge about the virus was still evolving and as new discoveries were made experts at times voiced contradicting opinions. People thus had to decide whether to vaccinate while they may have been uncertain on what the was best strategy (44). On the one hand, this gives an important role to social norms as cues for how to act (32). On the other hand, it is not evident that clear social norms of vaccination have had the chance to develop. We hypothesize that vaccine hesitancy is associated with inaccurate or weak empirical and/or normative expectations.

We conducted two studies (N=887 and N=412) among people living in Rome, with quasi-representative samples stratified by gender and age. In a cross-sectional study repeated every two weeks in the period June–August 2021, Study 1 measured vaccine acceptance rates, diagnosed whether a social norm of vaccination existed through incentivized elicitation of empirical and normative expectations, tracked if this norm changed over time, and assessed to what extent this norm suffered from biases or misperceptions. We show that particularly vaccine-hesitant people misperceive the prevailing social norm of vaccination (both in their empirical and in their normative expectations) and that misperceptions did not improve as vaccination rates increased. Based on the results of Study 1, Study 2 tested whether correct information about the social norm we may decrease vaccine hesitancy.

On August 6, 2021, once we reached 66% of our target sample for Study 2, the Italian government implemented a COVID-19 health certificate (the “green pass”) for travel and for many domestic activities (e.g. regulating entry to public venues, restaurants, cafes, bars, shops). Because this new regulation interfered with our norm-based interventions, we also explore how the green pass affected vaccine hesitancy and the social norm of vaccination. We explore whether the green pass effectively decreased vaccine hesitancy or, conversely, increased it (e.g. because of ethical, trust, and privacy concerns) (45–47). Second, the introduction of a new regulation can signal which behaviors are common or desirable in a group, so we explore whether the green pass shifted people’s perception about the social norm.

We contribute to existing literature on social norms and vaccination (and on social norm interventions in general) in two important ways. First, most research on social norms and vaccination decisions is correlational, meaning that while norms may drive vaccination intentions, it could as well be that those who want the vaccine are more likely to think that other people do as well (i.e. reverse causality) (30, 32). By comparing the results of the social norm interventions to a control group, our design rules out the reverse causality explanation.

Second, we propose a two-step approach of norm diagnosis and intervention. Norm diagnosis involves assessing for a relevant reference group the type of social norm in place (if any), how this norm develops over time, and whether it suffers from biases. Norm interventions use this diagnosis to present information about the norm to strengthen it and test whether the norm causally influences behavior (48, 49).

With some notable exceptions (31), most studies that test the effect of norm interventions on vaccination intentions skip the norm diagnosis and rely on hypothetical norms. They compare descriptive norm messages in which they vary the vaccination intentions in the population (24, 32) or they provide a general injunctive message that their peers think they should take the vaccine (28). This approach risks presenting subjects with implausible or irrelevant information, potentially rendering the intervention ineffective. More importantly, nothing is known about the starting conditions. It is unclear whether a social norm of vaccination exists, what this norm entails, how this norm develops, and whether it suffers from biases and misperception (50). If it is not a social norm, but personal convictions that drive decision-making, social norm interventions are unlikely to be successful, or may even lead to unintended effects (51, 52) (see, e.g. (53, 54) for examples of norm interventions that backfired).

## Study 1: Diagnosis of the vaccination norm over time

The large uncertainty surrounding how to behave in the pandemic gives a potentially large role for social norms in guiding these choices (55). However, it is reasonable to assume that when vaccines against COVID-19 first became available, the social norm of vaccination still had to develop (44). In our norm diagnosis, we therefore tracked how the norm developed over a period of two months.

Several factors potentially hindered the development of a social norm of vaccination. First, since vaccination generally is a private action, the main source through which people may update their social expectations about vaccine acceptance is through publicly reported vaccination rates. This information source may give a biased view of vaccine acceptance as long as not everyone has had the chance to get vaccinated. In Italy, the vaccination campaign started in January 2021, but the younger age groups were only invited to make a vaccination appointment by the end of June 2021. Second, overexposure of vaccine-hesitant people in traditional and social media may bias social expectations of vaccine acceptance in the actual population (56). Third, vaccine-hesitant people may wrongly perceive that few others want to get vaccinated due the well-known false consensus bias, according to which people overestimate how much others behave and think similarly to themselves (57).

There are indeed indications that people’s empirical expectations of how many people plan to get vaccinated against COVID-19 were lower than the reported vaccination intentions and their normative expectations (what other people approve of) lower than the reported beliefs about the importance of receiving the COVID-19 vaccine (30, 31). On the basis of this reasoning, we expect that social expectations of vaccine acceptance improve over time as actual vaccination rates increase, but that social expectations are lower than reported vaccine acceptance, and particularly so for vaccine-hesitant people.

### Methods

The data used for this study were collected through a cross-sectional survey that was conducted every two weeks from June 6 until August 1, 2021 among a quasi-representative sample from Qualtrics. All respondents lived in Rome (Italy) at the time of the study and the sample was stratified on age and gender (N=887, with $N_{1}=110$, $N_{2}=110$, $N_{3}=220$, $N_{4}=220$, $N_{5}=217$ respondents). To minimize differences between respondents in the extent to which they are informed about the latest COVID-19 developments, we presented all respondents with COVID-19 statistics on infection, mortality, and vaccination rates for the two preceding weeks (see Table S1 in Supplementary Materials). The survey then asked about vaccine acceptance and other COVID-related behavior and social norms, for which we asked respondents their social expectations about people living in Rome. Using a local reference population makes the norm more salient and simplifies the formulation of social expectations (52, 48).

Vaccine acceptance, personal normative beliefs, and empirical and normative expectations were measured by asking respondents to imagine a Person A who decides against receiving the vaccine against COVID-19. To measure vaccine acceptance (AC), we asked “Imagine that you are Person A and that you have been given the possibility to get vaccinated against the Coronavirus (COVID-19) with the vaccine of your choice. Would you have taken the vaccine?”. Respondents could answer “Yes, I already got vaccinated (1st and/or 2nd dose),” “Yes, I’ll get vaccinated when it is my turn,” “No, I don’t want to get vaccinated,” “I’m not sure if I want to get vaccinated.” The first two options were labeled as “vaccine accepting” (AC), the third and fourth as “vaccine hesitant.”

To measure empirical expectations (EE), we follow the method of (58) and asked “How many of the people from Rome that are taking part in this survey do you think would have decided to get the vaccine if they were invited to do so this week?” and explained that they should estimate the percentage of people participating in the same survey that answered “Yes” (I got the vaccine or I’ll get the vaccine) to the previous question in 10 categories from 0–10% to 90–100%. Measuring expectations this way reduces precision, but makes it easier for respondents to answer the question and should therefore reduce the tendency to answer through focal points (e.g. 0, 50, or 100)—a tendency observed, for instance, for empirical expectations reported in (31).

To measure personal normative beliefs (PNB) and normative expectations (NE), we combine the approaches of (58) (who incentivize NE based on the accuracy with respect to aggregate PNB) and (59) (who measure NE by asking for the modal appropriateness rating). Personal normative beliefs were measured by asking “How appropriate do you consider the decision of person A not to get vaccinated?.” The answers were measured on a 6-point Likert scale ranging from “extremely inappropriate” to “extremely appropriate.” Finally, to measure normative expectations respondents were asked to guess what would be the most frequent answer given to the previous question by people of Rome that are taking part in the same survey. Empirical and normative expectations were incentivized by rewarding the respondent whose estimates were closest to the aggregate reported answers with a bonus of €25.^a^ Misperceptions were calculated by subtracting for each respondent their expectation from that wave’s sample average (i.e. %AC_*t*_ − EE_*i,t*_ and PNB_*t*_ − NE_*i,t*_). For empirical expectations we report the misperception with respect to the midpoint of these categories (5 to 95%), but as a more conservative test we also offset reported vaccine acceptance to the upper limit of empirical expectations (seen as the minimum level of misperception that may be expected).

Control variables in the analyses were gender (female or not), age (18–84), nationality (Italian or not), perceived health, whether the respondent has been infected with COVID-19, how severe they perceive the risk of COVID-19, and the absolute change in the number of infections in Lazio (the region of which Rome is the capital) compared to the week before (see Table S3 in Supplementary Materials).

### Results

**Fig. 1.**
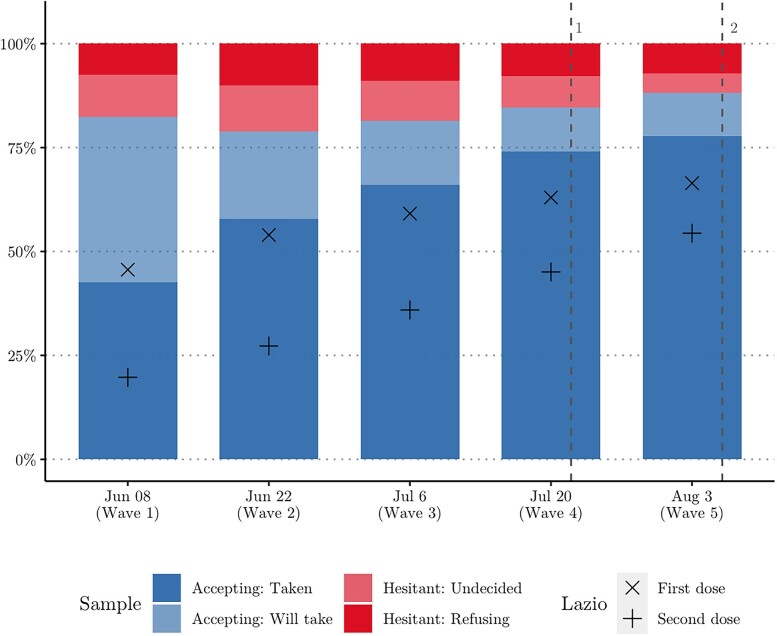
Vaccination trends over time in Lazio (the Italian region in which the capital Rome is located) and among survey respondents from Rome. *Notes:*  ^1^Green pass announced (July 22); ^2^Green pass implemented (August 6).

Population-wide vaccination rates in Lazio increased from 46 to 66% (first dose) over the study period. In the same period, 43 to 78% of the respondents (aged 18–84) reported having taken at least one dose of the vaccine (Fig. 1). The sample vaccination rate is slightly higher, because it includes adults only. However, it cannot be excluded that vaccination rates in Rome are higher than in Lazio overall, that vaccinated people are overrepresented in our study, or that social desirability influenced responses to the question.

Respondents that already were vaccinated are on average 10 years older than respondents who planned to get vaccinated. The groups are similar with respect to gender, nationality, perceived health, whether they have had COVID-19, and how they perceive the COVID-19 risk. The two categories will thus be treated as a single category of vaccine-accepting people. Among the hesitant respondents, those that self-identified as unsure about taking the vaccine are more often women ($m_{d}=0.24$) and perceive the COVID-19 risk to be lower ($m_{d}=-0.468$) than respondents that refuse vaccination. The two groups are similar with respect to age, nationality, perceived health, and COVID-19 sickness history. They each make up 8% of the total sample, so they are treated as a single category of 16% vaccine-hesitant people in the analyses.

In the first three waves, the percentage of vaccine accepting respondents (vaccinated + will vaccinate) was constant around 80%, indicating that the increase in official vaccination rates mostly follows from people that were already accepting of the vaccine. The official announcement of the COVID-19 green pass (a COVID-19 health certificate that gave access to public facilities) on July 2022 (when we collected Wave 4) led to an increase in the percentage of vaccine-accepting people to 85% in Wave 4 and 88% in Wave 5 ($\frac{\partial y}{\partial x}=0.020$, p=0.032).

**Fig. 2.**
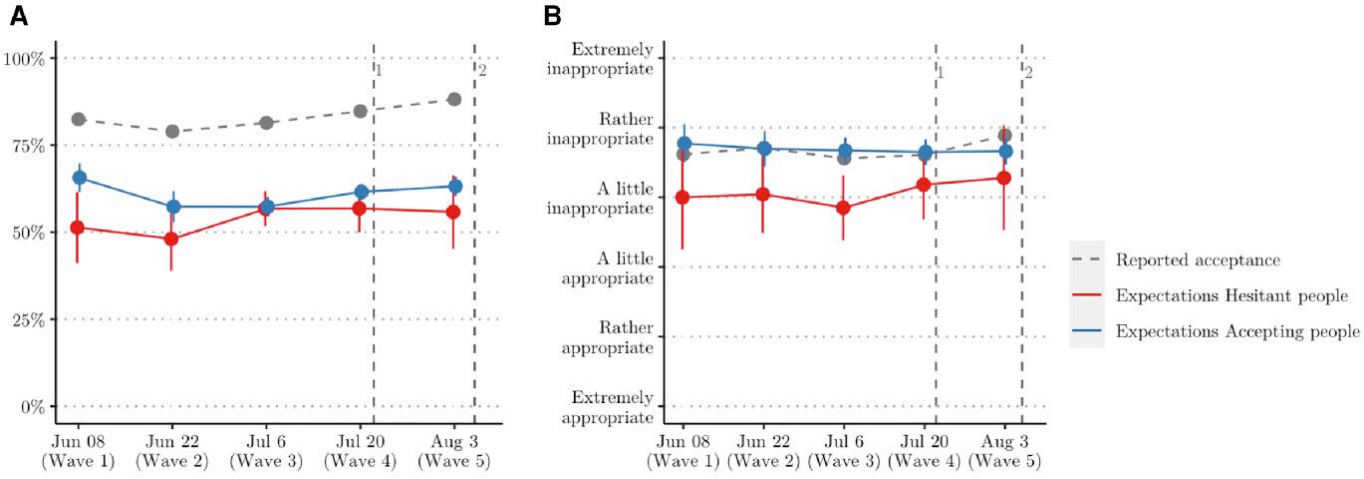
Social expectations for vaccine accepting and hesitant respondents over time. a) Empirical expectations of vaccine acceptance; b) normative expectations about the appropriateness of not vaccinating.

*Notes:*  ^1^Green pass announced (July 22); ^2^Green pass implemented (August 6)

Figure 2 reports vaccine acceptance and empirical expectations about vaccine acceptance (panel a) as well as normative beliefs and normative expectations about the inappropriateness of not vaccinating (panel b) for vaccine accepting (blue line) and vaccine-hesitant people (red line). Both accepting and hesitant people strongly underestimated how many people in Rome are willing to get vaccinated, with a significantly higher misperception for vaccine-hesitant people (b=0.07, p<0.001, $\eta^{2}=0.02$, see Table 1). Over the whole study period, the average vaccine acceptance in Rome was 84%. Vaccine accepting respondents had average empirical expectations of 61% and thus underestimated vaccine acceptance by 23%. Vaccine-hesitant respondents, on the other hand, on average reported empirical expectations of 53%. In other words, they expected only a small majority of the population to be vaccine accepting, and misperceived vaccine acceptance by as much as 30%. If we were to use the upper limit of the empirical expectations as a more conservative test of misperception, the differences with the reported vaccine acceptance remain substantial. The minimum misperception of vaccine-accepting respondents is 18% and that of vaccine-hesitant respondents 25%. Normative beliefs are misperceived only by vaccine-hesitant people (b=0.616, p<0.001, $\eta^{2}=0.03$). The normative expectations of vaccine-accepting people are in line with the average reported normative beliefs (b=.002, p=0.961).

**Table 1.** OLS regression on estimation bias in empirical and normative expectations.

	Empirical expectations				Normative expectations			
	b	se	b	se	b	se	b	se
Constant	0.229${}^{***}$	(0.007)	0.155${}^{*}$	(0.064)	0.002	(0.049)	−0.192	(0.438)
Vaccine hesitant${}^{a}$	0.070${}^{***}$	(0.017)	0.057${}^{**}$	(0.018)	0.613${}^{***}$	(0.115)	0.517${}^{***}$	(0.122)
Wave			0.018${}^{***}$	(0.005)			0.101${}^{**}$	(0.036)
Female			0.048${}^{***}$	(0.013)			0.056	(0.091)
Age			0.001	(0.000)			−0.007${}^{*}$	(0.003)
Italian			−0.042	(0.039)			−0.048	(0.272)
Perceived health			−0.012	(0.006)			−0.041	(0.043)
COVID-19 infection			0.028	(0.024)			−0.203	(0.163)
Perceived risk			0.022${}^{*}$	(0.010)			0.146${}^{*}$	(0.066)
Change tot. infections			−0.000	(0.000)			−0.000	(0.000)
*N*		877		867		877		867
$R^{2}$		0.020		0.055		0.031		0.048

Note: ${}^{*}p<0.05$, ${}^{**}p<0.01$, ${}^{***}p<0.001$.

${}^{a}$ Reference category: vaccine-accepting people.

Contrary to our expectations, misperception did not decrease as region-wide vaccination rates went up. If anything, misperception increased (although the effect sizes are small; $b_{ee}=0.018$, p=0.001, $\eta^{2}=0.013$; $b_{ne}=0.018$, p=0.001, $\eta^{2}=0.013$). Figure 2 suggests that this is due to increases in vaccine acceptance (panel a) and normative beliefs (panel b) that were not matched by changing expectations.

Finally, women misperceive empirical expectations more (b=0.101, p=0.006, $\eta^{2}=0.009$), younger people misperceive normative expectations more (b=−0.007, p=0.035, $\eta^{2}=0.005$), and people that perceive the COVID-19 risk as more severe misperceive both empirical (b=0.022, p=0.010, $\eta^{2}=0.002$) and normative expectations more (b=0.146, p=0.028, $\eta^{2}=0.006$).

### Generalizability

Altogether, a weak social norm of vaccination was diagnosed in the sense that people expect a slight majority of others to get vaccinated and expect that not vaccinating is considered inappropriate. The norm clearly suffers from misperceptions which could be potentially mitigated if people were informed about the actual acceptance and approval rates. However, these results pertain to a specific geographic location and may not be informative of norms and vaccine hesitancy in other contexts. Do other populations in other periods also underestimate vaccine acceptance? To assess the generalizability of our results, we compared them to data from two other data sources.

Data from the MIT COVID-19 beliefs survey conducted in 67 countries demonstrate that while both vaccine acceptance and empirical expectations about vaccine acceptance vary widely across countries, in all countries people on average underestimate the degree of vaccine acceptance by at least 10% between October 2020 and March 2021 (60). Using the aggregate data about Italy from the MIT COVID-19 beliefs survey, we calculated an average misperception between estimated and reported vaccine acceptance of 9% between December 27, 2020 (the start of the vaccination program) and March 15, 2021 (see Fig. S1 in the Supplementary Materials). Again, the degree of misperception did not change much over the study period. On March 15 (about three months before our study), the reported vaccine acceptance in Italy was 82% and the empirical expectations (of vaccine-accepting and vaccine-hesitant people combined) were 75% (i.e. an average misperception of 7%). It should be noted that in this case empirical expectations were asked with respect to the respondent’s local community whereas vaccine acceptance rates were taken from country-wide population statistics (31). If local vaccination rates differ from country-wide vaccination rates, this may bias the estimated misperception.

As a second comparison, we used the Periscope survey (11), which contains information about vaccine acceptance and empirical expectations in Bulgaria, France, Italy, Poland, Spain, and Sweden between June 14 and June 25, 2021, a period that overlaps with the first two waves of our survey. Calculating the misperception in empirical expectations for these countries, we find significant misperceptions for all countries (See Fig. S2 in the Supplementary Materials). However, while the empirical expectations of people in most countries underestimated vaccine acceptance (ranging from misperceptions of 2.5% in Sweden to 20.9% in Poland), Bulgarians actually overestimated the reported vaccine acceptance by 21.1%.

Combined, these results suggest that the COVID-19 vaccination norm often suffered from misperceptions. At least with respect to the descriptive norm people underestimated the actual vaccine acceptance rates. These data sources did not contain information about normative expectations. Moreover, while a general pattern of social norm misperceptions is established in early stages of the vaccination campaign, it is yet to be determined whether correcting this misperception would also influence vaccine hesitancy.

## Study 2: Norm-based messages to decrease vaccine hesitancy

Results of Study 1 signal that there were strong misperceptions about vaccine acceptance and vaccine appropriateness during the initial vaccination rollout period for vaccine-hesitant people, which did not decrease as vaccination rates increased. The high vaccination acceptance and appropriateness rates reported by the respondents indicate that there is an important information gap. Study 2 uses norm-based interventions to correct these misperceptions. Norm-based interventions aim to change behavior by providing information about the social norm (48, 49). This way, we speed up the process of norm formation and test the causal effect of norms on behavior. If the norm-based messages are effective, this means that biased social expectations partially explain hesitancy towards vaccination.

Interventions could correct empirical expectations, normative expectations, or both (see (61)) for a discussion of how empirical and normative information might affect also other types of beliefs (e.g. factual beliefs, personal normative beliefs). As discussed in the Introduction, we expect interventions that provide informational feedback both on empirical and normative expectations to be more effective than interventions that act on one of them separately. Generating the expectation that the majority of others follows a vaccine norm may not be a sufficient reason to conform, because it may tap on the temptation to free-ride on the efforts of others or it may reduce the perceived risk of infection (37, 40), which justifies the decision to wait. If high empirical expectations generates the believe that the overall COVID-19 risk goes down, this reduces the need to get vaccinated immediately, and allows vaccine-hesitant people to postpone their decision and see if vaccinated people suffer from any side effects.

On the other hand, interventions to strengthen the normative belief that the majority thinks one should get vaccinated without resolving the strategic uncertainty about the behavior of others (42, 41) may generate inconsistency between normative and empirical expectations when people still hold the empirical expectation that many people will not get the vaccine. Such an intervention may be ineffective because, for instance, vaccine-hesitant people do not expect that violations of the normative belief would be punished. Instead, when interventions simultaneously boost the expectations that a norm is largely followed and that the majority finds this the socially appropriate thing to do, normative and empirical expectations work in the same direction and motivate people to comply with the social norm.

Because the “green pass” (a COVID-19 health certificate that restricted access to public places) was introduced during our data collection, we also explore how this affected both misperceptions about the social norm of vaccination and vaccine hesitancy.

### Methods

Study 2 was conducted through Qualtrics between July 27 and August 29, 2021 with a quasi-representative sample stratified by gender and age from Qualtrics. Respondents were screened based on their vaccination choice at the beginning of the study. Only respondents that answered “No, I don’t want to receive the vaccine” (hereafter labeled as “Refusing”) or “I’m not sure if I want the vaccine” (hereafter labeled as ‘Undecided’) could participate in the study. Of the 3303 people living in Lazio that responded to the survey, 448 people (14%) met the vaccination selection criterion (212 refused vaccination and 236 were undecided). A second screening to match the age and gender stratification criteria resulted in a final sample size of N=412 respondents (N=192 vaccine refusing and N=220 undecided).

At the start of the survey, respondents were asked the same questions on social expectations as in Study 1. Subsequently, respondents were randomly assigned to one of the treatments with different norm-based messages (complete text in Table 2). The control treatment (Treatment 1) saw only the Introduction text (“Now the final questions about vaccines against COVID-19”). The other three treatments saw the Introduction, the Main message, and one or both of the Manipulation texts. Respondents assigned to Treatment 2 would get the norm-based message targeting empirical expectations, respondents to Treatment 3 the message targeting normative expectations, and respondents assigned to Treatment 4 saw the complete text, including both the empirical and the normative message.

**Table 2.** Elements of norm-based message presented to respondents of the four treatments.

Item	Text	1	2	3	4
Introduction	Now the final questions about vaccines against COVID-19.	x	x	x	x
Main	From a previous study, conducted between June 9 and July 7, 2021, we know that in Rome the percentage of people in favor of vaccination against COVID-19 is high.		x	x	x
Empirical	**81%** of the people expressed the wish to get vaccinated or already received the vaccine (one or two doses).		x		x
Normative	**76%** of the people consider the behavior of someone who decides not to get vaccinated between slightly and extremely inappropriate.			x	x

After presenting the norm-based message, vaccine hesitancy was measured using five items from the Oxford COVID-19 Vaccine Hesitancy scale (62) (see Table S4 in Supplementary Materials). An example item is “I would describe myself as …” with answer categories ranging from “Eager to get a COVID-19 vaccine” to “Anti-vaccination for COVID-19.” The higher the score, the more the respondent is vaccine hesitant.

A total of 66% of our target sample (N=271) responded to our survey before the green pass was introduced. Of these respondents, 139 was undecided and 132 was vaccine refusing. Through random assignment, they were equally divided over the four treatments. Another 141 respondents (79 undecided and 62 refusing) participated after the introduction of the green pass on August 6, 2021. While they were likewise equally divided over the four treatments, the small sample size does not permit comparisons of the treatment effects by vaccine hesitancy category after the introduction of the green pass.

### Results

**Fig. 3.**
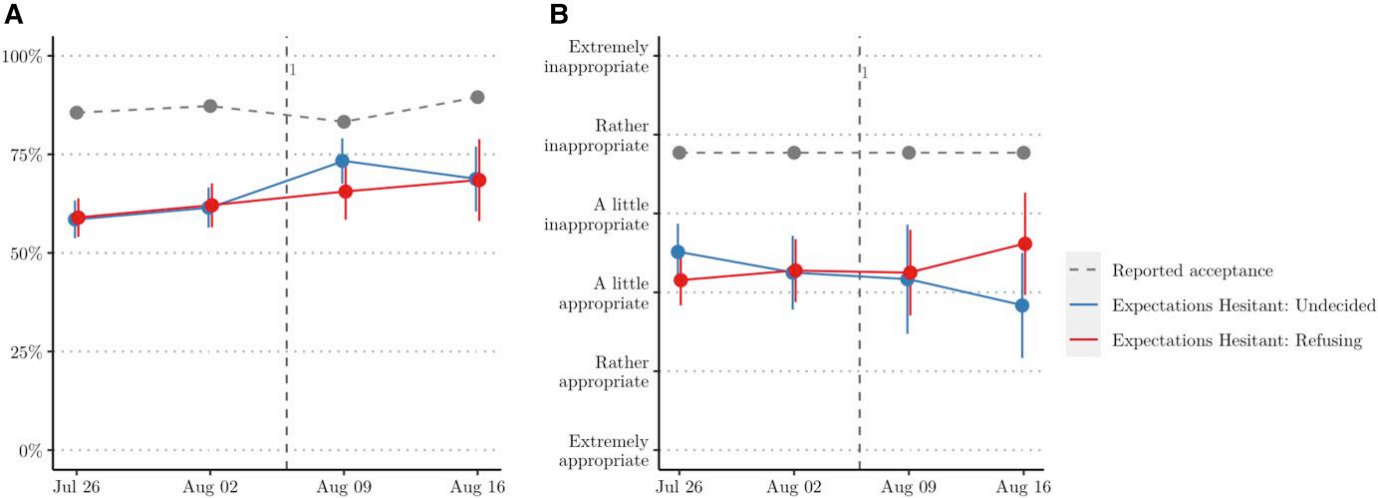
Social expectations for vaccine-hesitant and refusing respondents over time. a) Empirical expectations of vaccine acceptance; b) normative expectations about the appropriateness of not vaccinating.

*Notes:*  ^1^Green pass implemented (August 6).

In the absence of norm misperception, the designed social norm intervention would be ineffective. Before analyzing the treatment effect we thus assessed whether in Study 2 people likewise misperceived vaccine acceptance and appropriateness (see Fig. 3 and Supplementary Tables S5 and S6). Overall, misperceptions remain substantial both for vaccine acceptance ($m_{d}=0.24$, p<.001, Cohen’s δ=1.07) and appropriateness ($m_{d}=0.96$, p<.001, Cohen’s δ=0.64). However, the misperception in empirical expectations did decrease in the second half of the study period ($m_{d}=0.10$, p<.001, Cohen’s δ=0.44) after the introduction of the green pass. The empirical expectations were 59% before the introduction of the green pass and increased to 69% afterwards. This decreased the misperception in empirical expectations from 27% to 17%—or from 22% to 12% if we take the more conservative upper limit of the empirical expectations. Hence, while in terms of statistical significance empirical expectations continue to misperceive vaccine acceptance, the perceived descriptive norm of vaccine acceptance substantially does not differ much from reported vaccine acceptance. People expect a large majority of others to be accepting of COVID-19 vaccination.

The introduction of the green pass did not affect the normative expectations about the appropriateness of getting vaccinated ($m_{d}=0.032$, p=0.851), nor did it change the personal normative beliefs about vaccine appropriateness of our respondents ($m_{d}=0.160$, p=0.362). Hence, the green pass only strengthened the descriptive component of the social norm of vaccination but not the injunctive part. In doing so, it did confound the manipulation of treatments 2 and 4 that aimed to correct misperceptions in empirical expectations. We therefore control for the health certificate in our analyses by comparing vaccine hesitancy of subjects (both undecided and refusing) in the different treatments before and after its introduction.

Table 3 shows the effects of the norm-based treatments and the introduction of the green pass on vaccine hesitancy (M1), their interaction with the vaccine hesitancy category (undecided or refusing) (M2), and the interaction between green pass and norm-based messages for the two hesitancy categories (M3). Since our dependent variable (vaccine hesitancy) is not normally distributed particularly for vaccine refusing subjects (their modal response is on the extreme end of the scale, see Figs. S3 and S4 in Supplementary Materials), we estimate all models with bootstrapped standard errors (1000 repetitions) to address non-normality and reduce the role of outliers. There are no overall reductions in vaccine hesitancy for any of the norm-based messages ($b_{T2}=-0.130$, p=0.353; $b_{T3}=-0.116$, p=0.392; $b_{T4}=-0.225$, p=0.088) compared to the control treatment, nor did the green pass significantly reduce vaccine hesitancy (b=−0.066, p=0.611).

**Table 3.** OLS regression on vaccine hesitancy with bootstrapped standard errors (N=401, 1000 reps).

	M1		M2		M3	
	b	se	b	se	b	se
Constant	5.669${}^{***}$	(0.506)	5.664${}^{***}$	(0.503)	5.731${}^{***}$	(0.517)
TM2: Empirical^1^	−0.130	(0.140)	−0.118	(0.177)	−0.242	(0.189)
TM3: Normative^1^	−0.116	(0.135)	−0.143	(0.159)	−0.152	(0.180)
TM4: Empirical + Normative^1^	−0.225	(0.132)	−0.256	(0.161)	−0.446${}^{*}$	(0.196)
Green pass	−0.066	(0.130)	−0.351${}^{*}$	(0.151)	−0.543${}^{*}$	(0.269)
Vaccine refusing^2^	0.551${}^{***}$	(0.110)	0.315	(0.223)	0.290	(0.241)
T2 × Refusing			−0.027	(0.298)	−0.068	(0.339)
T3 × Refusing			−0.032	(0.286)	−0.024	(0.328)
T4 × Refusing			0.090	(0.287)	0.249	(0.338)
Green pass × Refusing			0.672${}^{**}$	(0.223)	0.659	(0.528)
T2 × Green pass					0.352	(0.442)
T3 × Green pass					−0.023	(0.361)
T4 × Green pass					0.489	(0.360)
T2 × Green pass × Refusing					0.297	(0.739)
T3 × Green pass × Refusing					0.121	(0.673)
T4 × Green pass × Refusing					−0.376	(0.687)
Female	0.185	(0.101)	0.237${}^{*}$	(0.102)	0.248${}^{*}$	(0.106)
Age	0.004	(0.004)	0.005	(0.004)	0.005	(0.004)
Italian	−0.353	(0.257)	−0.342	(0.246)	−0.332	(0.262)
Perceived health	−0.074	(0.053)	−0.070	(0.054)	−0.071	(0.055)
COVID-19 infection	0.201	(0.173)	0.208	(0.169)	0.195	(0.173)
Perceived COVID-19 risk	−0.692${}^{***}$	(0.070)	−0.693${}^{***}$	(0.068)	−0.693${}^{***}$	(0.069)
Change total infections	0.000	(0.000)	0.000	(0.000)	0.000	(0.000)
*N*		401		401		401
LR $\chi^{2}$ difference			10.49${}^{*}$	(4)	5.40	(6)
$R^{2}$	0.344		0.361		0.369	

Note: ${}^{*}p<0.05$, ${}^{**}p<0.01$, ${}^{***}p<0.001$.

${}^{1}$ Reference category: Treatment 1.

${}^{2}$ Reference category: Undecided respondents.

We do find a large difference in the reported vaccine hesitancy of vaccine refusing respondents compared to undecided respondents (b=0.551, p<0.001, $\eta^{2}=0.068$). In Model M2, we see this difference translated in a significant interaction effect of the green pass with the vaccine hesitancy category (b=0.672, p=0.003, $\eta^{2}=0.025$). Substantially, this means that the introduction of the green pass has significantly reduced vaccine hesitancy for undecided respondents (b=−0.351, p=0.020). For vaccine refusing respondents, on the other hand, vaccine hesitancy does not change significantly (b=0.322, p=0.090). Note that this reflects a between-subjects comparison. Respondents that self-classify as not wanting the vaccine before the introduction of the green pass report the same levels of hesitancy as those who self-classify as refusing after the introduction of the green pass. Respondents that self-classify as unsure before the introduction of the green pass, however, where more hesitant than those who self-classified as unsure after. While we do not have this information, it is not unreasonable that the result may be underestimated: people who initially self-classified as unsure may have responded to want to get vaccinated after the introduction of the green pass, which means they were no longer selected into our sample.

**Fig. 4.**
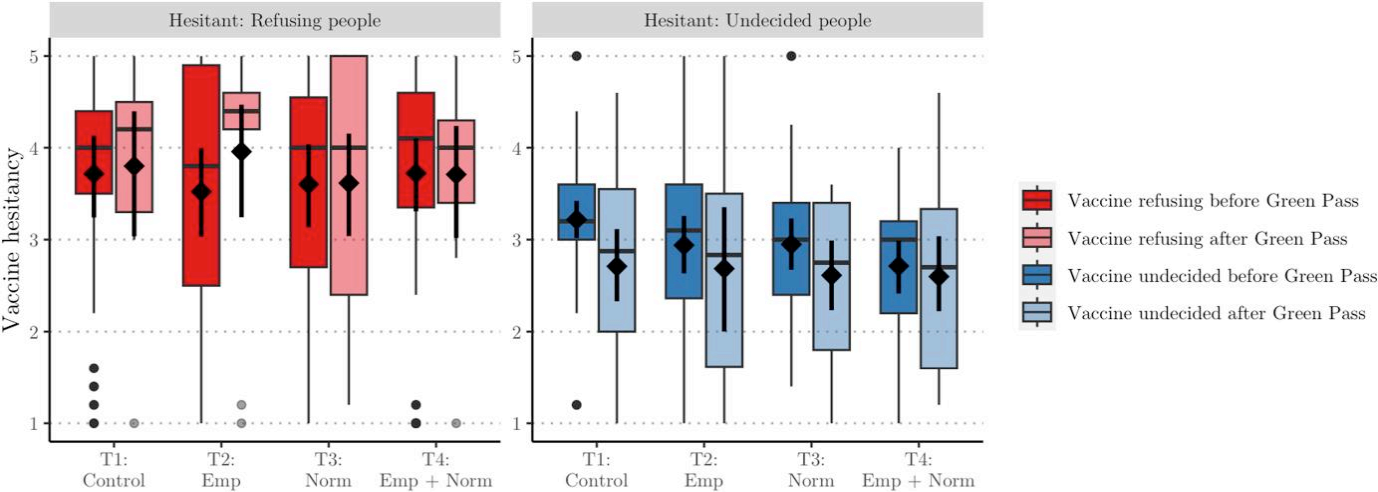
Mean, bootstrapped confidence intervals, and boxplot distributions of mean hesitancy per treatment for two vaccine categories before and after implementation of the green pass.

The norm-based messages had no effect neither for undecided respondents ($b_{T2}=-0.118$, p=0.503; $b_{T3}=-0.143$, p=0.368; $b_{T4}=-0.256$, p=0.111) nor for vaccine refusing respondents ($b_{T2}=-0.146$, p=0.534; $b_{T3}=-0.175$, p=0.451; $b_{T4}=-0.166$, p=0.474). To explore why the norm treatments have had no effect we plot in Fig. 4 the boxplot distribution of vaccine hesitancy for each treatment split by refusing (left panel) and undecided respondents (right panel), and before and after the green pass was implemented. The distributions in the left panel clearly indicate that the norm-based messages have had no effect on the vaccine refusing subjects. If anything, we see an increased heterogeneity, where some report lower hesitancy and others higher.

For the undecided respondents in the right panel, however, there is a tendency towards decreasing hesitancy following empirical, normative, or empirical and normative messages. In fact, the results of Model M3 suggest that before the introduction of the green pass, treatment 4 (correcting both empirical and normative expectations) significantly reduced vaccine hesitancy for undecided respondents (b=−0.446, p=0.023, $\eta^{2}=0.003$). That would imply that the green pass, by decreasing hesitancy in all treatments including the control, nullified the effect of the norm-based message. However, the increased complexity introduced by the three-way interaction does not significantly improve the model compared to Model M2 (LR $\chi^{2}(6)=5.40$, p=0.493). The sample size lacks the statistical power to reliably estimate this final model, which casts limitations on the robustness of this result.

## Conclusion

We presented a method of social norm diagnosis and intervention to identify whether vaccine hesitancy is partially social driven. That is, we tested whether a social norm of vaccination exists, how it developed over time, whether it suffers from misperceptions, and whether vaccination decisions are conditional on the social norm. Most studies either only measure the norm in place (29, 30) (assuming this norm to be stable) or manipulate a hypothetical norm to test its causal effect on behavior (32). The first approach identifies a norm, but cannot conclude whether this norm is stable or still evolving, nor whether the norm drives behavior or rather the reverse. The second approach is weak in terms of external validity, because it is unknown what drives the effect (if any) and how it translates to the real dynamic norm in place. The two-step approach of diagnosis and intervention, instead, provides accurate and credible information and controls the causal relation between norms and behavior (48, 52).

We found severe misperceptions in the social norm of vaccination. All respondents (both vaccine accepting and vaccine hesitant) underestimated empirical expectations and this did not change as vaccination rates increased, which suggests that the vaccination norm, which had to be build from scratch, was slow to develop spontaneously. Vaccine-hesitant people misperceived empirical expectations more strongly than vaccine-accepting respondents. Moreover, their normative expectations also underestimated the real normative beliefs. This misperception may indicate a false consensus bias: the tendency to assume that others are similar to oneself. Using different data sources, we replicated the finding of an underestimation in vaccine acceptance for different populations and across different time periods (11, 60), thus generalizing our findings beyond the local context of Rome.

Given these low social expectations, it is reasonable to assume that the (weak) social norm in place did little to motivate vaccine-hesitant people. Presenting them with norm-based messages that reveal the accurate social information corrects their social expectations and may potentially decrease their hesitancy. While we observe some decreases in the vaccine hesitancy reported by respondents that self-identify as unsure about getting vaccinated, we do not find overall support for this hypothesis. It is plausible that the COVID-19 certificate that was introduced during our data collection nullified the effect of norm-based messages by decreasing both vaccine hesitancy and misperceptions in the empirical expectations.

Similar to earlier studies (46, 47, 45), we find an interaction between regulations and social norms and observe that the introduction of the green pass affected the perception of the descriptive norm of vaccine acceptance. Before its introduction there was a large gap between the empirical expectations and the vaccine acceptance. The empirical expectations were closer to the vaccination rates publicly available on the media and used in many COVID-19 communication campaigns. The data suggest that in this period providing information correcting both empirical and normative expectations reduced hesitancy for vaccine-hesitant respondents, although it should be kept in mind that the small sample size makes the analyses underpowered.

The introduction of the green pass, necessary for traveling and many other indoors and outdoors activities, led people to increase their expectations about how many others would be willing to get the vaccine, if only to avoid such restrictions. This has decreased the misperception and as such has left little space for our norm-based messages to be effective. On the other hand, differently from (46, 47, 45) the green pass did not decrease misperceptions in the normative expectation. Hence, even after the introduction of the green pass there was no strong (injunctive) social norm of vaccination.

Of course, certain limitations need to be kept in mind. First, because we did not anticipate the introduction of the green pass during our data collection, the analyses of Study 2 were underpowered. There are indications that before the introduction of the green pass providing information about both the descriptive and the injunctive component of the norm might have been successful in reducing vaccine hesitancy, but this result may not be robust. Moreover, while we attempted to provide more accurate information by diagnosing the norm in place first, it cannot be excluded that the observed vaccine acceptance rates are inflated due to social desirability bias in the survey response. The social expectations questions were incentivized, but the questions about vaccination intentions and personal normative beliefs were not. Moreover, we have no way to know whether the norm-based messages resulted in an updating of the social norm or rather induced some sort of experimenter demand effect. Finally, we do not know if changes in vaccine hesitancy translate into people getting vaccinated. That is, from the current study set-up it cannot be concluded that social norms can be leveraged to increase vaccination rates.

However, altogether the method of diagnosis and interventions allowed us to identify social norms, their internal dynamics and their (potential) effect in driving attitudes and behavior with more precision. This way, the two studies illustrate practical applications about how social norms can be mapped and leveraged in vaccination campaigns. Since early misperceptions about vaccine acceptance correlate highly with the publicly reported vaccination rates, the detailed updates about actual vaccination rates may have hindered the development of a social norm of vaccination. If we want a social norm of vaccination in early stages of a vaccination campaign, it seems important to not only report on current vaccination rates, but also on vaccination intentions and beliefs about vaccination appropriateness in a population.

## Supplementary Material

pgad132_Supplementary_Data

## Data Availability

The data and scripts underlying this article are available on OSF at https://osf.io/xp2h6.

## Note

a See Table S2 in Supplementary Materials for precise question wording.

## Previous Presentation

These results were previously presented at the conference of the Italian Association of Cognitive Sciences (AISC) in Noto (Italy) on December 13–15, 2021.
