# Correction to: Vaccine-hesitant people misperceive the social norm of vaccination

**DOI:** 10.1093/pnasnexus/pgad218

**Published:** 2023-07-04

This is a correction to: Eva Vriens and others, Vaccine-hesitant people misperceive the social norm of vaccination, PNAS Nexus, Volume 2, Issue 5, May 2023, pgad132, https://doi.org/10.1093/pnasnexus/pgad132

In the originally published version of this manuscript, the equation in the following sentence in the Methods section was incorrect:

Misperceptions were calculated by subtracting for each respondent their expectation from that wave's sample average (i.e. EE_i,t_ − %AC_t_ and NE_i,t_ − PNB_t_).

The sentence has been corrected to read as follows:

Misperceptions were calculated by subtracting for each respondent their expectation from that wave's sample average (i.e. %AC_t_ − EE_i,t_ and PNB_t_ − NE_i,t_).

