# Correction to: Vaccine-hesitant people misperceive the social norm of vaccination

**DOI:** 10.1093/pnasnexus/pgad366

**Published:** 2023-11-15

This is a correction to: Eva Vriens, Luca Tummolini, Giulia Andrighetto, Vaccine-hesitant people misperceive the social norm of vaccination, *PNAS Nexus*, Volume 2, Issue 5, May 2023, pgad132, https://doi.org/10.1093/pnasnexus/pgad132

During a retroactive audit conducted by *PNAS Nexus*, it was discovered that this paper was missing a statement acknowledging compliance with the *PNAS Nexus* Human and Animal Participants and Clinical Trials policy:

Ethical approval was obtained from the ethical board of the Institute of Cognitive Sciences and Technologies of the Italian National Research Council (AMMCNT-CNR n. 0065527/2019). Participants in our studies were informed about the data collection, data storage, and their individual rights and provided informed consent before participating.

This error has been corrected in the original article.

