# Supplementary material for: Vaccine-hesitant people misperceive the social norm of vaccination
Source: PNAS Nexus. 2023 Apr 15;2(5):pgad132. doi: 10.1093/pnasnexus/pgad132 (PMC10165803; doi:10.1093/pnasnexus/pgad132)
Supplement: pgad132_Supplementary_Data [file pgad132_supplementary_data.pdf]

Supplementary materials to "Vaccine hesitant people misperceive the social norm of vaccination"

Eva Vriens[a,b,*], Luca Tummolini[a,b], and Giulia Andrighetto[a,b,c]

[a]Institute of Cognitive Sciences and Technologies, Italian National Research Council, Rome, Italy
[b]Institute for Futures Studies, Stockholm, Sweden
[*]To whom correspondence should be addressed: eva.vriens@istc.cnr.it

Contents

S1 Supplementary materials to Study 1 **2**
S1.1 Methods . . . 2
S1.2 Generalizability . . . 5

S2 Supplementary materials to Study 2 **7**
S2.1 Methods . . . 7
S2.2 Results . . . 8

List of Figures

S1 Difference between vaccine acceptance and empirical expectations in Italy from October 29 2020 to March 15 2021 . . . 5
S2 Difference between vaccine acceptance and empirical expectations of vaccine hesitant people in six European countries in June 2021 . . . 6
S3 Distribution of vaccine hesitancy items per treatment and vaccination category . . . 9
S4 Distribution of vaccine hesitancy scale per vaccination category . . . 10

List of Tables

S1 Information presented to respondents about the latest COVID-19 developments. . . . 2
S2 Norm measurement questions. . . . 3
S3 Descriptive statistics Study 1 . . . 4
S4 Items in the vaccine hesitancy scale. . . . 7
S5 Descriptive statistics Study 2 . . . 8
S6 OLS regression on estimation bias in empirical and normative expectations (Study 2). . . . 8

S1 Supplementary materials to Study 1

S1.1 Methods

For Study 1, the same survey was conducted among a new stratified sample of respondents living in Rome (Italy) every two weeks between June 6 and August 1, 2021. To minimize differences between respondents in the extent to which they were informed about recent Covid-19 developments, we provided information about recent Covid-19 developments (infections, dates, and vaccinations) at the beginning of the survey (see Table S1). A dataset with the dates and numbers that were used in each wave is available on OSF at https://osf.io/xp2h6.

Table S1: Information presented to respondents about the latest COVID-19 developments.

**Cases**	On **June 6** in Lazio the total number of infections since the start of the COVID-19 pandemic was **343,435** (**6%** of the population). **8223** people have died because of the coronavirus (COVID-19). From **May 31** until **June 6** **1412** new positive cases were reported. **48** people have died. One week earlier the number of cases was higher. From **May 24** to **May 30** there were **2119** new cases and **74** deaths.
**Vaccinations**	On **June 6** in Lazio, **3,707,219** vaccines have been administered, calculated since December 27 2020. **46%** of the population has received the first dose of the vaccine against the Coronavirus (COVID-19). **19%** is completely vaccinated (**1,102,384** people have received both the first and the second dose). One week earlier, on **May 30**, **41%** of the population had received the first dose of the vaccine and **17%** (**976,984** people) were completely vaccinated.

Note: Dates and numbers in bold were updated at the start of a new wave.

To diagnose norms, we asked about individual behavior, empirical expectations, personal normative beliefs, and normative expectations (see Table S2). The questions about empirical and normative expectations were incentivized. The person whose estimate to all of the social expectations questions in the survey was closest to the reported behaviors and beliefs earned an additional 25 Euro.

Table S2: Norm measurement questions.

Item	Answer options
Imagine a person, Person A, who despite being invited to receive the Covid-19 vaccine has chosen not to take it.	
Imagine being Person A and being given the possibility to get vaccinated against Covid-19 with the vaccine of your chose. Would you have taken the vaccine?	1 Yes, I already got vaccinated<br>2 Yes, I will get vaccinated when it is my turn<br>3 No, I do not want the vaccine<br>4 I am not sure whether I want the vaccine<br>5 I prefer not to answer[1]
How appropriate do you find the decision of Person A not to get vaccinated?	1 Extremely inappropriate<br>2 Rather inappropriate<br>3 Slightly inappropriate<br>4 Slightly appropriate<br>5 Rather appropriate<br>6 Extremely appropriate
How many people in Rome participating in the survey like you do you think would get the vaccine? We ask you to make an estimate of how many people have answered Yes to the previous question (Yes, I already got vaccinated or Yes, I will get vaccinated when it is my turn).<br><br>*The estimate - if accurate - allows you to earn more money from participating in this survey. The participant that is most accurate in his/her estimates to this and the other questions obtains an additional 25 Euro.*	1 0–10%<br>2 10–20%<br>3 20–30%<br>4 30–40%<br>5 40–50%<br>6 50–60%<br>7 60–70%<br>8 70–80%<br>9 80–90%<br>10 90–100%
According to you, what is the most frequent answer given by people in Rome participating in this survey like you have given to the question "How appropriate do you find the decision of Person A not to get vaccinated?"<br><br>*We ask you to make an estimate that - if accurate - allows you to earn more money from participating in this survey. The participant that is most accurate in his/her estimates to this and the other questions obtains an additional 25 Euro.*	1 Extremely inappropriate<br>2 Rather inappropriate<br>3 Slightly inappropriate<br>4 Slightly appropriate<br>5 Rather appropriate<br>6 Extremely appropriate

Note: [1] 'I prefer not to answer' was coded as missing.

In the analyses we controlled for gender (female or not), age, nationality (Italian or not), perceived health, whether the respondent has been infected with Covid-19 in the past, the perceived risk of Covid-19, and the change in the number of infections in Lazio compared to the week before (as reported at the start of the survey). Perceived health was measured asking respondents to rate their personal health on a scale from 1 ('Very poor') to 6 ('Very healthy'). The perceived risk was measured through four items: 'The Coronavirus (Covid-19) will affect very many people in Rome', 'I will probably get sick with the Coronavirus', 'The Coronavirus is dangerous', and 'The current infection rate of the Coronavirus scares me'. Respondents could indicate their answer on a five-point Likert scale from 1 'Strongly disagree' to 5 'Strongly agree'. The four items had an acceptable Cronbach's α reliability ($\alpha = 0.695$) and we took the average to create a single perceived risk scale. Finally, we controlled for the difference in the number of infections in that week compared to the week before as communicated to respondents at the beginning of the survey (Table S1). See Table S3 for the summary statistics of all variables used in the analyses.

Table S3: Descriptive statistics Study 1

	N	M	SD	Min	Max
Estimation bias empirical expectations	877	0.24	0.19	−0.16	0.83
Estimation bias normative expectations	877	0.11	1.33	−1.44	3.89
Vaccine hesitant	877	0.18		0	1
Female	876	0.51		0	1
Age	877	42.56	14.64	18	84
Italian	877	0.97		0	1
Perceived health	874	4.84	1.06	1	6
Covid-19 infection	870	0.08		0	1
Perceived risk	877	2.68	0.72	1	5
Change tot. infections	877	173.52	847.87	−707	1606

S1.2 Generalizability

To test the external validity and generalizability of our results, we compared the findings to those of two other cross-country data collections. Data from the MIT Covid-19 beliefs survey conducted in 67 countries[1] demonstrate that while both vaccine acceptance and empirical expectations about vaccine acceptance vary widely across countries, in all countries worldwide people on average underestimate the degree of misperception by at least 10% between October 2020 and March 2021 (see [1]).

Using their aggregate data for Italy[2], we estimated the misperception between estimated and reported vaccine acceptance in Italy for this period (see Figure S1). Before the vaccination program started (from October 20 to December 7), empirical expectations correctly estimated nation-wide vaccine acceptance (EE = 63%, acceptance = 62%). As soon as the vaccination program starts, empirical expectations underestimate vaccine acceptance. On December 27 2020, at the start of the vaccination program, acceptance jumped to 72.4% while empirical expectations were still 63.8% (i.e., an average misperception of 8.6%). By March 15 2021, acceptance was 81.4% (similar to the 82% on June 8 2021 in our results) and expectations 74.5% (reflecting a misperception of 6.9%). While the empirical expectations are higher than the 63% reported by people in Rome on June 8 2021 in our survey, the misperception is substantial over the full study period also using this data source.

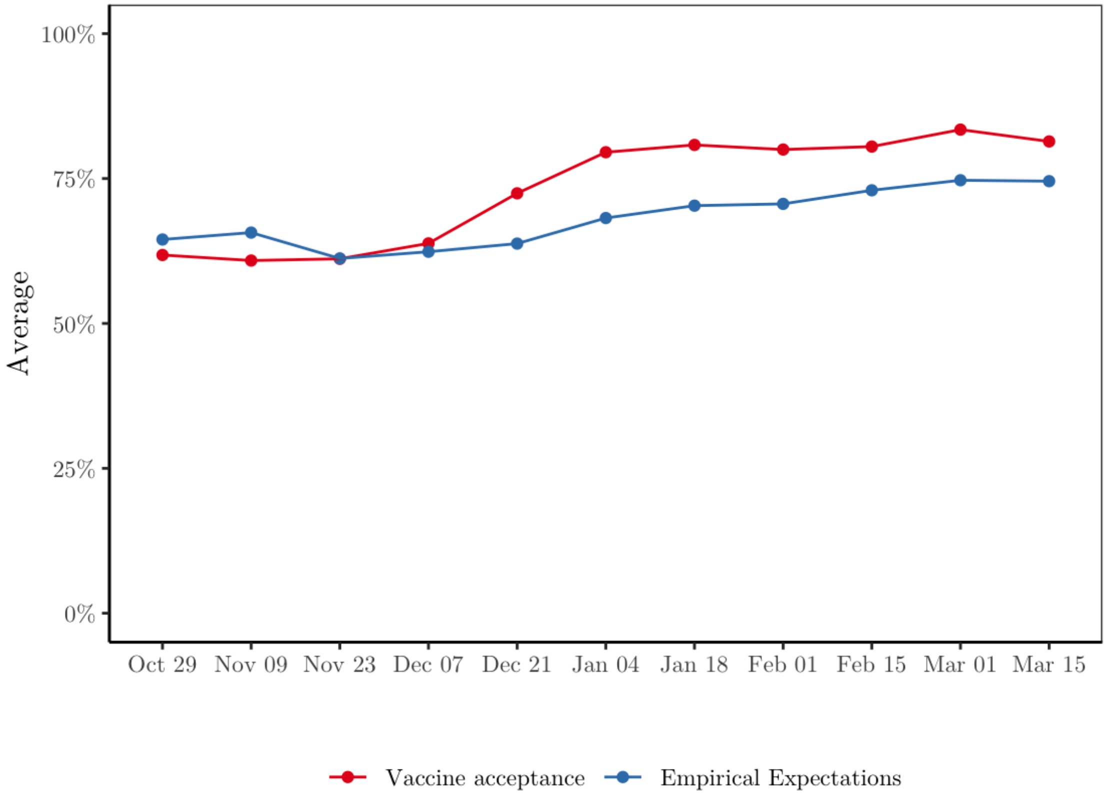

Figure S1: Difference between vaccine acceptance and empirical expectations in Italy from October 29 2020 to March 15 2021.
Source: MIT Covid-19 Beliefs Survey based on N = 29162 respondents over 11 waves in Italy.

[1] Countries included are: Afghanistan, Algeria, Angola, Argentina, Australia, Azerbaijan, Bangladesh, Bolivia, Brazil, Cambodia, Cameroon, Canada, Cote d'Ivoire, Chile, Colombia, Ecuador, Estonia, Egypt, France, Georgia, Germany, Ghana, Guatemala, Honduras, Indonesia, India, Iraq, Italy, Jamaica, Japan, Kazakhstan, Kenya, Malaysia, Mexico, Mongolia, Morocco, Mozambique, Myanmar, Nigeria, Netherlands, Nepal, Pakistan, Peru, Philippines, Poland, Portugal, Romania, Senegal, Singapore, South Africa, South Korea, Spain, Sri Lanka, Sudan, Taiwan, Tanzania, Thailand, Trinidad & Tobago, Turkey, Uganda, Ukraine, United Arab Emirates, United Kingdom, United States, Uruguay, Venezuela, Vietnam

[2] This data is downloaded from `https://covidsurvey.mit.edu`

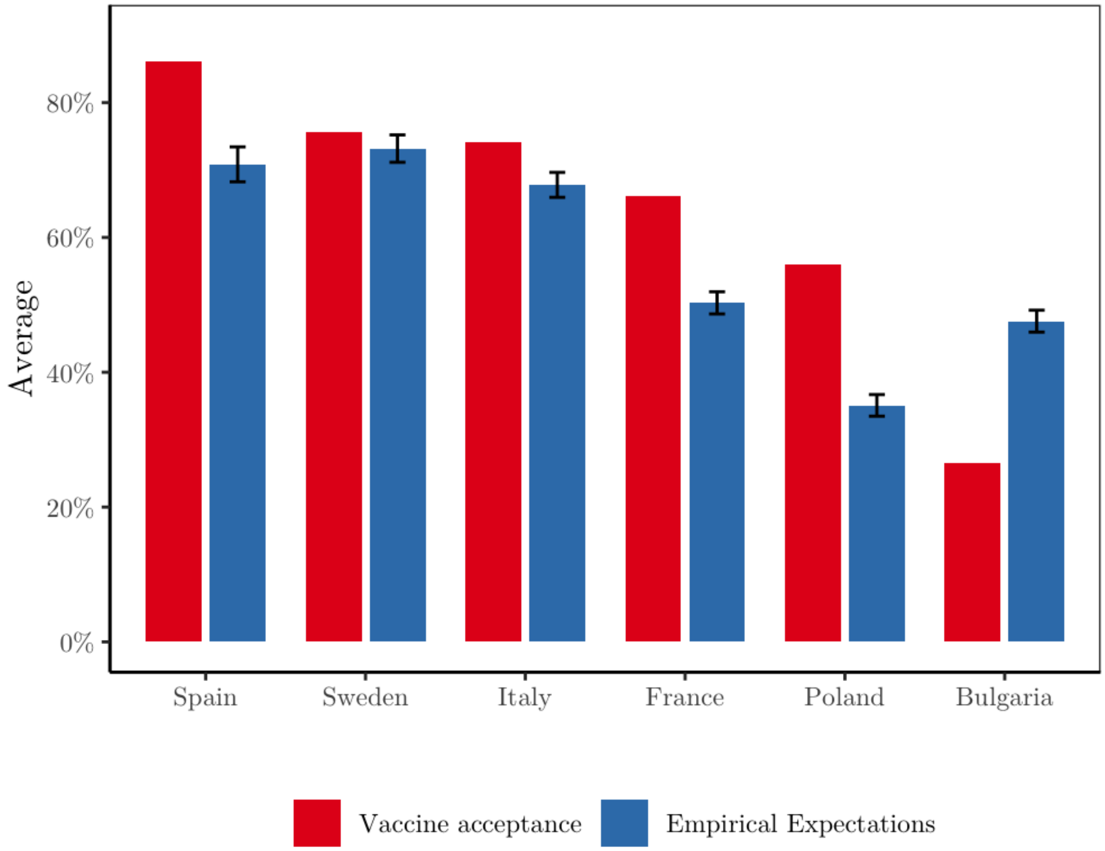

Figure S2: Difference between vaccine acceptance and empirical expectations of vaccine hesitant people in six European countries in June 2021.
Source: Periscope data, based on survey on Covid-19 attitudes and beliefs in Bulgaria (N = 964), France (N = 943), Italy (N = 744), Poland (N = 990), Spain (N = 380), and Sweden (N = 618) collected between June 15 and June 24 2021.

The second data source is the Periscope Survey[3], that collected information about vaccine acceptance and empirical expectations of acceptance in Bulgaria, France, Italy, Poland, Spain, and Sweden in June 2021 (around the same time as Waves 1 and 2 of our Study 1). We estimated the misperception between aggregate acceptance and individual empirical expectations for each of the six countries (see Figure S2. We find significant underestimation in the empirical expectations of vaccine acceptance for Poland (% accepting = 56.0%, EE = 35.1%, misperception = 20.9, $p < 0.001$), France (% accepting = 66.1%, EE = 50.3%, misperception = 15.8, $p < 0.001$), Spain (% accepting = 86.2, EE = 70.8, misperception = 15.3, $p < 0.001$), Italy (% accepting = 74.2%, EE = 67.8%, misperception = 6.4, $p < 0.001$), and Sweden (% accepting = 75.7, EE = 73.2, misperception = 2.5, $p = 0.014$). For Bulgaria, on the other hand, we find a significant overestimation, which is due to the low overall vaccine acceptance (% accepting = 26.5%, EE = 47.6%, misperception = -21.1, $p < 0.001$).

S2 Supplementary materials to Study 2

S2.1 Methods

In Study 2, $N = 192$ vaccine refusing and $N = 220$ undecided people were randomly assigned to one of four treatment conditions. After reading one of four norm-based messages, they had to answer five questions of the Oxford Covid-19 Vaccine Hesitancy scale (Table S3).[2] The 'Don't Know' category was coded as missing. The five items had a very high Cronbach's α reliability ($\alpha = 0.951$). We used the average of the five items to create a vaccine hesitancy scale.

Table S4: Items in the vaccine hesitancy scale.

Item	Answer options
I would describe my attitude towards receiving a COVID-19 vaccine as	1 Very keen<br>2 Pretty positive<br>3 Neutral<br>4 Quite uneasy<br>5 Against it<br>6 Don't know
If my family or friends were thinking of getting a COVID-19 vaccination, I would	1 Strongly encourage them<br>2 Encourage them<br>3 Not say anything to them about it<br>4 Ask them to delay getting the vaccination<br>5 Suggest that they do not get the vaccination<br>6 Don't know
I would describe myself as	1 Eager to get a COVID-19 vaccine<br>2 Willing to get the COVID-19 vaccine<br>3 Not bothered about getting the COVID-19 vaccine<br>4 Unwilling to get the COVID-19 vaccine<br>5 Anti-vaccination for COVID-19<br>6 Don't know
Taking a COVID-19 vaccination is	1 Really important<br>2 Important<br>3 Neither important nor unimportant<br>4 Unimportant<br>5 Really unimportant<br>6 Don't know
Would you take a COVID-19 vaccine if offered?	1 Definitely<br>2 Probably<br>3 I may or I may not<br>4 Probably not<br>5 Definitely not<br>6 Don't know

Note: Since the original scale was developed before vaccines were publicly available, two items that asked what respondents would do if the vaccine would be available were excluded from our survey.

In testing for treatment differences we used the same control variables as in Study 1 (gender, age, nationality, perceived health, history of Covid-19 infection, perceived risk, and change in the number of infections. For the latter, we used information of new Covid-19 infections during the four weeks of the data collection and took the number of cases in week 1 of Study 2 as a baseline (Table S4).

Table S5: Descriptive statistics Study 2

	N	M	SD	Min	Max
Vaccine hesitancy	411	3.24	1.19	1	5
Vaccine hesitant: Undecided	412	0.53		0	1
Female	410	0.51		0	1
Age	412	39.73	13.02	18	83
Italian	412	0.96		0	1
Perceived health	408	5.13	0.98	1	6
Covid-19 infection	407	0.11		0	1
Perceived risk	412	2.95	0.85	1	5
Change tot. infections	412	−136.25	486.20	−1224	328

S2.2 Results

Before testing for treatment differences, we checked whether the estimation bias still existed by repeating the same OLS regression analyses that we ran also for Study 1 (Table S5). This is the case for both empirical and normative expectations, but non of the control variables explain this bias for the vaccine hesitant sample.

Table S6: OLS regression on estimation bias in empirical and normative expectations (Study 2).

	Empirical Expectations				Normative expectations			
	b	se	*b*	se	*b*	se	*b*	se
Constant	0.232***	(0.016)	0.176	(0.117)	−1.064***	(0.118)	−0.270	(0.891)
Refusing[1]	0.008	(0.022)	0.015	(0.023)	0.032	(0.162)	0.122	(0.173)
Green pass			−0.078**	(0.030)			0.011	(0.225)
Female			−0.004	(0.023)			0.252	(0.171)
Age			0.000	(0.001)			−0.002	(0.007)
Italian			−0.012	(0.055)			−0.118	(0.418)
Perceived health			0.009	(0.012)			−0.060	(0.092)
Covid-19 infection			0.007	(0.036)			0.199	(0.273)
Perceived risk			−0.002	(0.014)			−0.120	(0.106)
Change tot. infections			0.052	(0.039)			−0.105	(0.293)
N		412		402		412		402
R^2		0.000		0.053		0.000		0.011

Note: * $p < 0.05$, ** $p < 0.01$, *** $p < 0.001$; [1] Reference category: undecided people.

As a robustness check, finally, we checked whether the treatment effects were consistent regardless of the hesitancy item used (see Figure S4. The effect was replicated for items 3, 4, and 5 of the vaccine hesitancy scale, but the difference between treatment 1 and 2 was not significant for items 1 and 2.

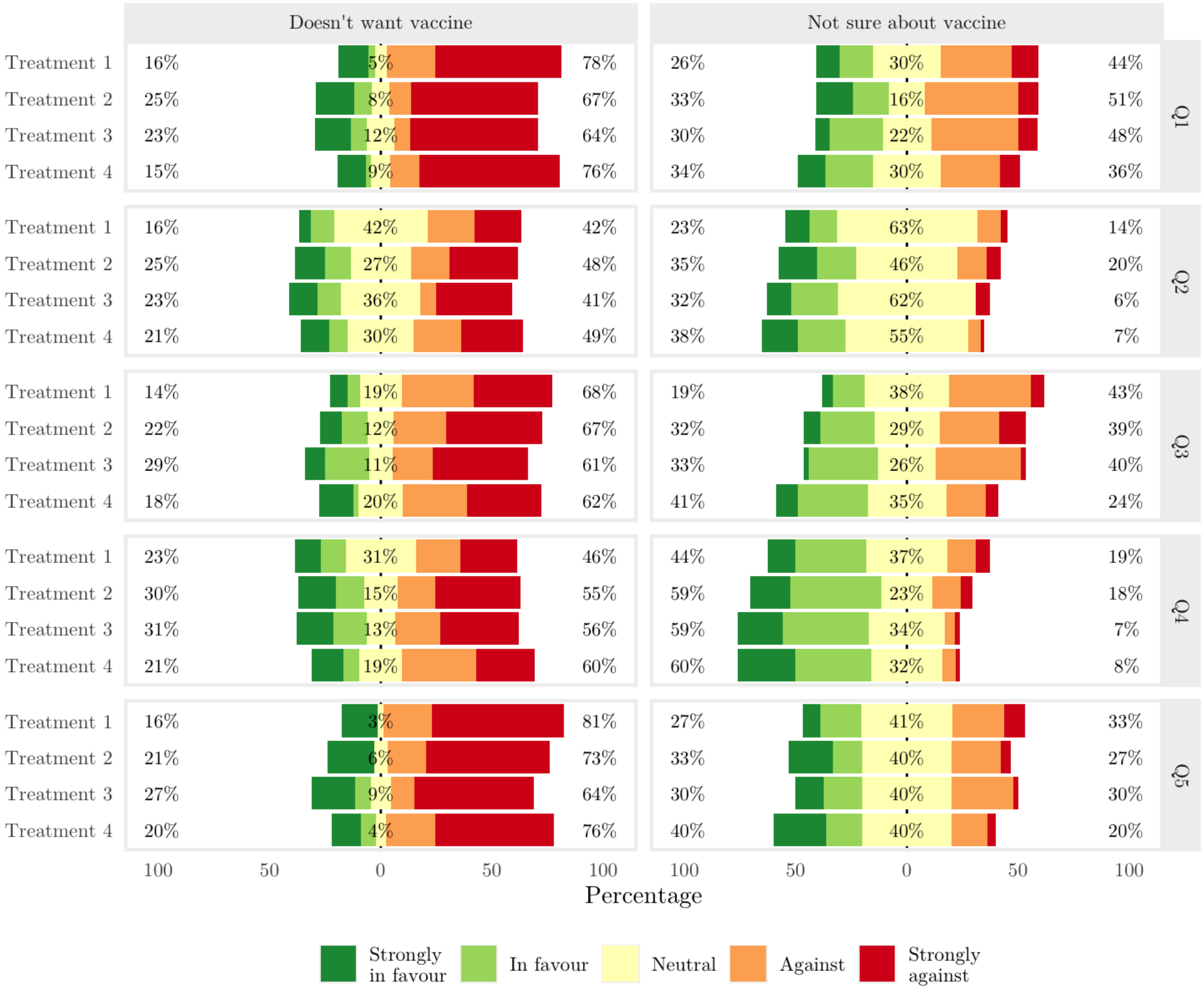

Figure S3: Distribution of vaccine hesitancy items per treatment and vaccination category

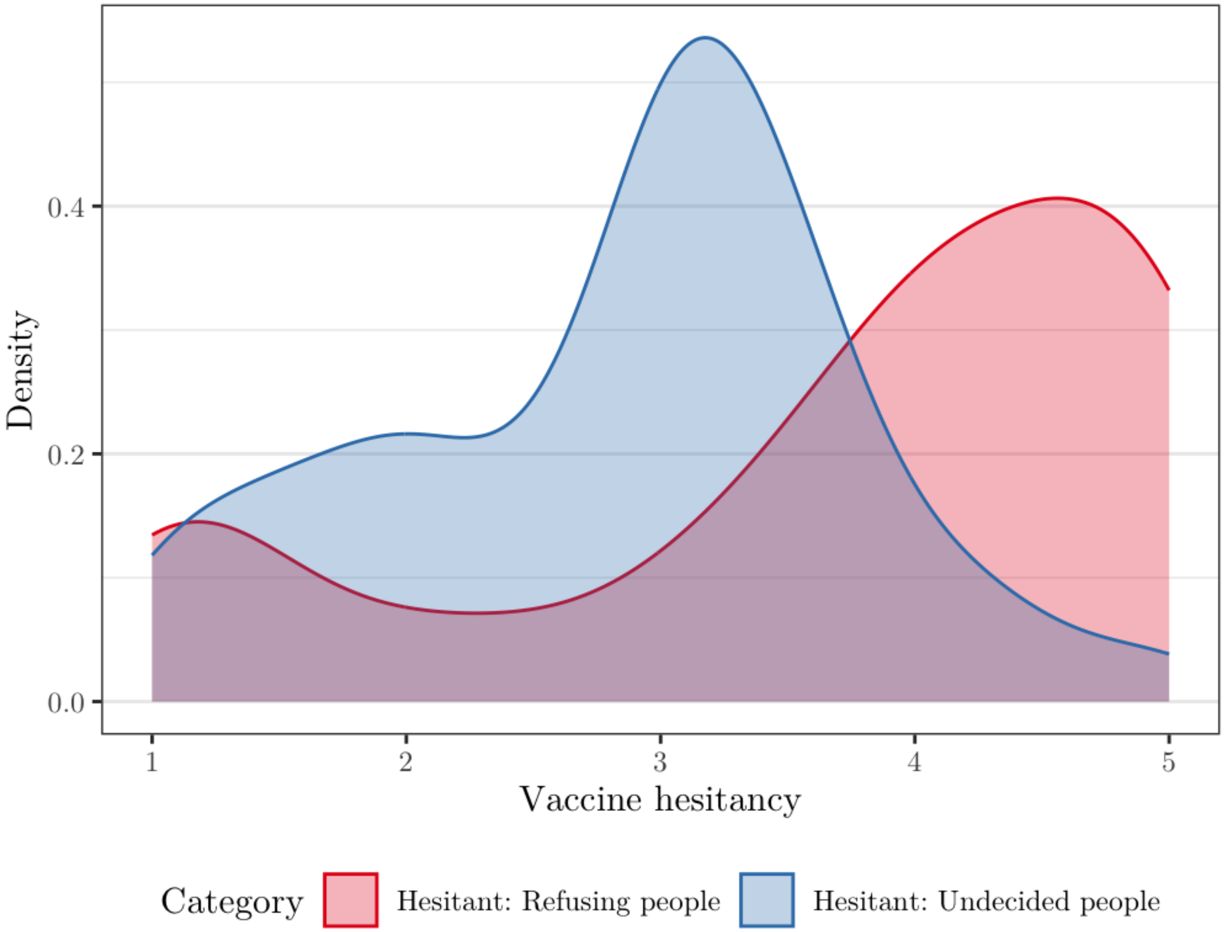

Figure S4: Distribution of vaccine hesitancy scale per vaccination category

References

[1] Avinash Collis, Kiran Garimella, Alex Moehring, M. Amin Rahimian, Stella Babalola, Nina H Gobat, Dominick Shattuck, Jeni Stolow, Sinan Aral, and Dean Eckles. Global survey on COVID-19 beliefs, behaviors, and norms. *Nature Human Behaviour*, (November):1–25, 2022.

[2] Daniel Freeman, Bao S. Loe, Andrew Chadwick, Cristian Vaccari, Felicity Waite, Laina Rosebrock, Lucy Jenner, Ariane Petit, Stephan Lewandowsky, Samantha Vanderslott, Stefania Innocenti, Michael Larkin, Alberto Giubilini, Ly Mee Yu, Helen McShane, Andrew J. Pollard, and Sinéad Lambe. COVID-19 vaccine hesitancy in the UK: The Oxford coronavirus explanations, attitudes, and narratives survey (Oceans) II. *Psychological Medicine*, 2021.

[3] Janina Steinert, Henrike Sternberg, Hannah Prince, Barbara Fasolo, Matteo Galizzi, Tim Buthe, and Giuseppe Veltri. COVID-19 Vaccine Hesitancy in Eight European Countries: Prevalence, Determinants and Heterogeneity. *Science Advances*, 8:1–15, 2022.
